# Association between ATN profiles and mortality in a clinical cohort of patients with cognitive disorders

**DOI:** 10.1186/s13195-023-01220-x

**Published:** 2023-04-10

**Authors:** Mélina Régy, Aline Dugravot, Séverine Sabia, Elodie Bouaziz-Amar, Claire Paquet, Bernard Hanseeuw, Archana Singh-Manoux, Julien Dumurgier

**Affiliations:** 1 Université Paris Cité, Inserm U1153, Epidemiology of Ageing and Neurodegenerative Diseases, Paris, France; 2 Université Catholique de Louvain, Brussels, Belgium; 3 Division of Psychiatry, Faculty of Brain Sciences, University College London, London, UK; 4 Université Paris-Cité, Department of Biochemistry, GHU APHP Nord Lariboisière Fernand-Widal Hospital, Paris, France; 5 Université Paris-Cité, Inserm U1144, Cognitive Neurology Center, GHU APHP Nord Lariboisière Fernand-Widal Hospital, Paris, France; 6 Cliniques Universitaires Saint-Luc, Brussels, Belgium

**Keywords:** Alzheimer’s disease, ATN classification, Mortality

## Abstract

**Background:**

Alzheimer’s disease (AD) is the 5th leading cause of death in people 65 years and older. The ATN classification reflects a biological definition of AD pathology with markers of Aβ deposition (A), pathologic tau (T), and neurodegeneration (N). Little is known about the relationship between ATN status and the risk of mortality, leading us to examine this association in a relatively large population of patients seen at a memory clinic for cognitive disorders.

**Methods:**

Data were drawn from the BioCogBank Study, including patients seen for cognitive disorders in Lariboisiere Hospital (Paris, France), followed up to 15 years. All participants underwent a lumbar puncture for an assessment of the levels of CSF tau (tau), phosphorylated tau (p-tau181), and β-amyloid 42 peptide (Aβ42). Vital status on July 1, 2020, was recorded for each participant using the national mortality register. Individuals were categorized according to their ATN profiles based on CSF Aβ42 or Aβ42/40 ratio, p-tau181, and tau. Kaplan–Meier and multivariate Cox analyses were performed with A-T-N − participants as the reference using a short (5 years) and long follow-up (15 years).

**Results:**

Of the 1353 patients in the study (mean age: 68 years old, 53% of women, mean MMSE score: 22.6), 262 died during the follow-up. At 5 years of follow-up, A-T-N + individuals had the highest risk of mortality in Kaplan–Meier and adjusted Cox analyses [HR (95% CI) = 2.93 (1.31–6.56)]. At 15 years of follow-up, patients in the AD spectrum had a higher mortality risk with a gradient effect for biomarker positivity: A-T + [HR = 1.63 (1.04–2.55)], A + T − [HR = 2.17 (1.44–3.26)], and A + T + individuals [HR = 2.38 (1.66–3.39)], compared to A-T-N − patients. Adjustments on potential confounders had little impact on these associations.

**Conclusion:**

This study shows ATN profiles to be associated with mortality in a relatively large patient cohort based on a memory clinic. Patients with isolated evidence of neurodegeneration had a higher mortality rate in the short follow-up, and patients with the AD profile had the highest mortality rate in the long follow-up.

**Supplementary Information:**

The online version contains supplementary material available at 10.1186/s13195-023-01220-x.

## Background

Alzheimer’s disease (AD) is the leading cause of dementia, affecting around 50 million of people [1]. Due to the global aging of the population, this number is expected to triple by 2050 [2]. Alzheimer’s disease is associated with lower life expectancy and is the 5th leading cause of death in developed countries for people aged 65 and older [3].

Neuropathology of AD is characterized by the presence of extracellular plaques of beta-amyloid peptides (Aβ) and neurofibrillary tangles constituted by the intraneuronal aggregation of hyperphosphorylated tau protein [4]. Brain MRI, tau and amyloid PET, and cerebrospinal fluid (CSF) biomarkers are increasingly used in the clinical diagnosis of AD [5–7] to detect the presence of neurodegeneration, abnormal amyloid peptides, or pathologic tau proteins in the brain.

In 2018, the National Institute on Aging – Alzheimer’s Association (NIA-AA) proposed the move towards a biological and “unbiased” definition of AD, based on CSF and imaging biomarkers: the ATN classification [8]. The label “A” refers to Aβ plaques and is determined using cortical amyloid PET ligand binding or low CSF Aβ42; the label “T” stands for fibrillar tau, assessed by an elevated level of CSF phosphorylated tau (p-tau181) or cortical tau PET ligand binding; and the label “N” indicates neurodegeneration or neuronal injury, determined by CSF tau (tau), 18F-fluorodeoxyglucose PET hypometabolism, or atrophy on brain MRI. The combination of ATN yields 8 biological profiles underlying the physiopathology of the disease (A-T-N − , A-T-N + , A-T + N − , A-T + N + , A + T-N − , A + T-N + , A + T + N − , A + T + N +). Further recommendations from the International Working Group have emphasized the need for a clinical-biological diagnosis of AD, which requires beta-amyloid and tau biomarker positivity along with a clinical phenotype typically observed in AD [9].

Several studies have examined the association of ATN profiles with cognitive decline [10–13], AD dementia [14, 15], white matter lesions [16], and brain atrophy [11], but their association with mortality remains unclear. Little is known about the vital prognosis of ATN-ascertained AD, and the few studies that have investigated the association between ATN biomarkers and mortality are inconsistent [17, 18]. Accordingly, we used data from a relatively large cohort of patients from memory clinic, followed for up to 15 years, to examine the short- and long-term associations between ATN profiles and mortality. We also examined associations with mortality for each CSF biomarker.

## Methods

### Study population

Data are drawn from the BioCogBank Study, a monocentric memory clinic-based cohort of 1354 patients followed at the Lariboisiere Hospital memory clinic department (Paris, France) between 2006 and 2020; further details are available elsewhere [19, 20]. In a clinical setting, all patients were assessed for the presence of cognitive disorders. The work-up included a lumbar puncture with measures of CSF Aβ42, CSF Aβ40 (since 2012), CSF tau, and CSF p-tau181. The study was approved by the Ethical Committee of Paris University Hospitals. All participants signed an informed, written consent.

### CSF analysis and cutoffs

Patients underwent a lumbar puncture in the morning, between 9 a.m. and 12 p.m., after a period of fasting. CSF samples were collected in polypropylene tubes; this included the CML model TC10PCS from January 2008 to November 2012 and Sarstedt catalog no. 62.610.201 from December 2012 to July 2020. The measure of CSF Aβ40 was added to the list of CSF biomarkers, starting in September 2012. Cutoffs for CSF biomarkers were determined using a data-driven method as described previously [21]; period-specific cutoffs are shown in Supplementary Table S1.

### ATN classification

A + classification was defined as CSF Aβ42 level or Aβ42/40 ratio below the period-specific cutoff, T + classification as CSF p-tau181 level above the period-specific cutoff, and N + classification if CSF tau level was above the period-specific cutoff (Supplementary Table S1).

### Outcome measure

Mortality data were extracted from the national French national mortality register [22] until July 1, 2020.

### Covariates

Covariates were ascertained using patients’ medical records.

Sociodemographic variables included age at first consultation, sex, and level of education (categorized as no education to primary school, secondary to high school, baccalaureate or university degree).

Global cognition was assessed using the Mini-Mental State Examination (MMSE), concurrently to the measure of CSF biomarkers. Cognitive stage (cognitively unimpaired, mild cognitive impairment, dementia) was extracted from medical records concurrently to the measure of CSF biomarkers.

Cardiovascular risk factors included hypertension (systolic/diastolic blood pressure ≥ 140/90 mmHg or use of antihypertensive drugs), smoking status (non-smoker, never or former, current smoker), alcohol consumption (never, occasional, regular but non-heavy, heavy consumption: 8 or more drinks per week for a woman and 15 or more drinks per week for a man), dyslipidemia (medical record of dyslipidemia or use of lipid-lowering drugs), and diabetes (medical record of diabetes or use of anti-diabetic medication) were assessed during the patients’ first consultation at the memory clinic.

Apolipoprotein E (APOE) genotype was determined from venous blood. Genomic DNA was extracted from 200 μL of frozen blood using automated procedures and dedicated DNA purification kits (Maxwell 16, Promega, Madison, WI, USA). After amplification of the exon 4 of the APOE gene, the APOE allelic patterns were identified using denaturing high-performance system liquid chromatography (WAVE DNA fragment analysis system, Transgenomic, Omaha, NE, USA) with appropriate controls [18, 23].

### Statistical analysis

Characteristics of patients included in the study were described overall and as a function of ATN profile and mortality. Categorical variables were compared using *χ*^2^ tests and continuous variables using one-way ANOVA.

The analyses of the association between CSF biomarkers and mortality were carried out using Cox regression. Patients were followed from the date of first consultation in the memory clinic until their date of death or until the date of the end of the study (July 1, 2020), whichever came first. We first examined whether the association between AT profiles (A-T − , A-T + , A + T − , A + T +), and mortality risk was modified by the presence of neurodegeneration (“N” status) by testing the interaction between AT profiles and N status in a Cox proportional hazard model adjusted for age, sex, and level of education. The overall interaction was not significant (*p* for interaction = 0.53), but there was an indication of a statistically significant effect in the A-T − group (*p* = 0.03; stratified analysis in Supplementary Table S2), leading us to consider 5 profiles in the ATN classification: A-T-N − (reference group), A-T-N + , A-T + , A + T − , and A + T + in further analyses. Survival probabilities in each ATN group were assessed using a short (5 years) and long (15 years) follow-up with Kaplan–Meier estimators. Survival probabilities were compared using the log-rank test for Kaplan–Meier analyses. The median survival corresponds to the time the estimate curve reaches a survival probability 50% on the Kaplan–Meier plot. A Cox proportional hazard model was used to examine the association of ATN profile with mortality risk over a follow-up of 5 years and 15 years. The analysis was first adjusted for age and sex (model 1) and then further adjusted for APOE4 status (e4 carrier or not), MMSE score, level of education, smoking, hypertension, and dyslipidemia (model 2). Covariates selected for model 2 were those associated either with ATN profiles or with mortality in univariate analyses with a *p*-value lower than 0.20. We used multiple imputation procedures to impute missing values of covariates (APOE genotype, smoking status, dyslipidemia, hypertension, alcohol status, and diabetes) using chained Eqs. (20 imputations) including all available baseline predictors, mortality status, and the delay of follow-up (mi commands in STATA). The linear trend for the association between AT profiles (excluding A-T-N + profile) and mortality was tested by including a variable coded 0, 1, 2, and 3 for A-T-N − , A-T + , A + T − , and A + T + groups, respectively, as a continuous variable in a Cox proportional hazard model. Interaction terms of sex and cognitive stage with ATN groups were tested in separate analyses to examine whether the association between ATN profiles and mortality risk differed by sex and cognitive stage.

The association of each CSF biomarker with mortality was examined in separate Cox proportional hazard models. The CSF biomarkers (Aβ42, Aβ40, tau, p-tau181, and Aβ42/40 ratio) were log-transformed and standardized using period-specific data to take into account the change in measurement technique. The proportional hazard assumption was checked using the Schoenfeld residuals method.

Two-tailed values of *p* < 0.05 were considered statistically significant; statistical analyses were performed using Stata 15 (StataCorp LP, College Station, TX).

## Results

### Baseline characteristics of the study population

The present study included 1353 (53% women, mean (standard deviation (SD)) age = 68.2 (9.8) years) patients who underwent a lumbar puncture to determine CSF levels of Aβ42, tau, and p-tau181 between July 2006 and July 2020. CSF levels of Aβ40, added later to the study, were available in 980 of these participants. Characteristics of the study population are presented in Table 1. The mean (SD) MMSE score at biomarker assessment was 22.6 (5.4). Twenty-seven percent of the population were cognitively unimpaired, 40% were at a MCI stage, and 33% at a dementia stage. A total of 503 (37%) patients had an Alzheimer’s disease A + T + profile. Compared to the normal biomarkers profile (A-T-N − , *n* = 418, 31%), those with A + T + profile were older, more likely to be women, were APOE4 carriers, and had a lower MMSE score. There were no differences in education or cardiovascular risk factors across ATN profiles. Participant characteristics as a function of the eight ATN profiles are shown in Supplementary Table S3. Some biomarkers profiles were uncommon: A-T + N − (*n* = 16) and A + T + N − (*n* = 14), corresponding to 1.2% and 1.0% of the study sample, respectively.

**Table 1:** Characteristics of the study population

	Overall population	Missing	A/T/N profiles					
			A − /T − /N −	A − /T − /N +	A − /T +	A + /T −	A + /T +	
	*N* = 1353	*N* (%)	*N* = 418	*N* = 56	*N* = 162	*N* = 214	*N* = 503	*p* value
Age, mean (SD)	68.2 (9.8)	0	63.8 (10.9)	66.1 (9.1)	70.7 (8.2)	69.5 (9.4)	70.6 (8.3)	< 0.001
Women, *n* (%)	722 (53.4)	0	193 (46.2)	33 (58.9)	84 (51.9)	109 (50.9)	303 (60.2)	< 0.001
Education level, *n* (%)								
Low	370 (27.4)		103 (24.6)	9 (16.1)	47 (29.0)	64 (29.9)	147 (29.2)	
Intermediate	455 (33.6)		146 (34.9)	20 (35.7)	57 (35.2)	70 (32.7)	162 (32.2)	
High	528 (39.0)		169 (40.4)	27 (48.2)	58 (35.8)	80 (37.4)	194 (38.6)	0.45
MMSE score, mean (SD)	22.6 (5.4)	0	24.5 (4.1)	23.3 (5.8)	23.8 (4.4)	21.6 (6.1)	21.0 (5.8)	< 0.001
Cognitive stage, *n* (%)								
Cognitively unimpaired	362 (26.8)		155 (37.1)	17 (30.4)	53 (32.7)	55 (25.7)	82 (16.3)	
Mild cognitive impairment	538 (39.8)		184 (44.0)	26 (46.4)	66 (40.7)	73 (34.1)	189 (37.6)	
Dementia	453 (33.5)		79 (18.9)	13 (23.2)	43 (26.5)	86 (40.2)	232 (46.1)	< 0.001
APOE4 carriers, *n* (%)	511 (42.4)	148 (10.9%)	75 (20.3)	11 (22.9)	51 (33.3)	100 (55.0)	274 (60.5)	< 0.001
Heavy alcohol consumption,^a^ *n* (%)	87 (7.6)	215 (15.9%)	36 (10.3)	4 (8.9)	7 (5.2)	11 (6.3)	29 (6.7)	0.40
Smokers, *n* (%)	123 (10.5)	183 (13.5%)	41 (11.2)	8 (17.0)	12 (8.7)	20 (11.1)	42 (9.6)	0.51
Dyslipidemia, *n* (%)	387 (31.3)	115 (8.5%)	107 (28.1)	13 (26.5)	56 (37.3)	52 (27.7)	159 (33.8)	0.11
Hypertension, *n* (%)	562 (45.2)	102 (7.5%)	157 (40.7)	19 (38.8)	74 (50.0)	84 (44.4)	228 (48.3)	0.12
Diabetes mellitus, *n* (%)	191 (15.4)	114 (8.4%)	67 (17.5)	8 (16.3)	26 (17.3)	23 (12.2)	67 (14.3)	0.45

^a^Eight or more drinks per week for a woman and 15 or more drinks per week for a man

### Baseline characteristics according to mortality status

During a maximum follow-up of 15 years, 262 (19.4%) patients died; their baseline characteristics are presented in Table 2. Patients who died were mostly men (57.2% vs 44.1%, *p* < 0.001) and at baseline were older (71.8 (8.5) years vs 67.3 (9.9) years, *p* < 0.001) and had poorer MMSE scores (21.0 (6.0) vs 23.0 (5.2), *p* < 0.001). They also tended to have more cardiovascular risk factors, though the difference between mortality cases and non-cases was statistically significant only for hypertension. Patients who died during the follow-up were more likely to have an abnormal biomarker profile and had lower mean levels of CSF Aβ42, CSF Aβ42/40 ratio, and higher levels of CSF tau and p-tau181.

**Table 2:** Characteristics of the study population according to mortality status at the end of follow-up

	Death during follow-up			*p* value^a^
	No	At 5 years	At 15 years	
	*N* = 1091	*N* = 121	*N* = 262	
Age, year, mean (SD)	67.3 (9.9)	71.6 (8.7)	71.8 (8.5)	< 0.001
Women, *n* (%)	610 (55.9)	47 (38.8)	112 (42.8)	< 0.001
Education level, *n* (%)				
Low	291 (26.7)	39 (32.2)	79 (30.2)	
Intermediate	361 (33.1)	38 (31.4)	94 (35.9)	
High	439 (40.2)	44 (36.4)	89 (34.0)	0.17
MMSE score, mean (SD)	23.0 (5.2)	19.8 (6.0)	21.0 (6.0)	< 0.001
Cognitive stage, *n* (%)				
Cognitively unimpaired	326 (29.9)	11 (9.1)	36 (13.7)	
Mild cognitive impairment	418 (38.3)	51 (42.2)	120 (45.8)	
Dementia	347 (31.8)	59 (48.8)	106 (40.5)	< 0.001
APOE4 carriers, *n* (%)	414 (42.0)	35 (36.8)	97 (44.3)	0.53
Heavy alcohol consumption, *n* (%)^b^	66 (7.1)	11 (10.7)	21 (9.9)	0.32
Smokers, *n* (%)	92 (9.7)	14 (13.1)	31 (14.0)	0.06
Dyslipidemia, *n* (%)	298 (30.0)	46 (39.7)	89 (36.2)	0.06
Hypertension, *n* (%)	430 (43.1)	62 (53.9)	132 (53.4)	0.004
Diabetes mellitus, *n* (%)	146 (14.7)	25 (21.6)	45 (18.4)	0.15
A/T/N profiles, *n* (%)				
A − /T − /N −	374 (34.3)	25 (20.7)	44 (16.8)	
A − /T − /N +	46 (4.2)	8 (6.6)	10 (3.8)	
A − /T +	126 (11.6)	12 (9.9)	36 (13.7)	
A + /T −	163 (14.9)	24 (19.8)	51 (19.5)	
A + /T +	382 (35.0)	52 (43.0)	121 (46.2)	< 0.001
CSF biomarkers, pg/mL, mean (SD)				
Aβ42	782.3 (344.7)	616.5 (273.7)	590.0 (267.6)	≤ 0.001
Aβ40	11,950 (4931)	10,443 (4633)	11,885 (5839)	0.90
Aβ42/40 ratio	0.078 (0.051)	0.074 (0.039)	0.067 (0.036)	0.02
Tau	387.8 (264.9)	567.2 (361.5)	518.6 (322.5)	≤ 0.001
p-Tau181	61.1 (38.0)	78.0 (43.8)	78.6 (40.7)	≤ 0.001
p-Tau181/Aβ42 ratio	0.11 (0.11)	0.17 (0.14)	0.17 (0.13)	≤ 0.001

^a^No mortality versus mortality at 15 years

^b^Eight or more drinks per week for a woman and 15 or more drinks per week for a man

### ATN profiles and risk of mortality

**Fig. 1:**
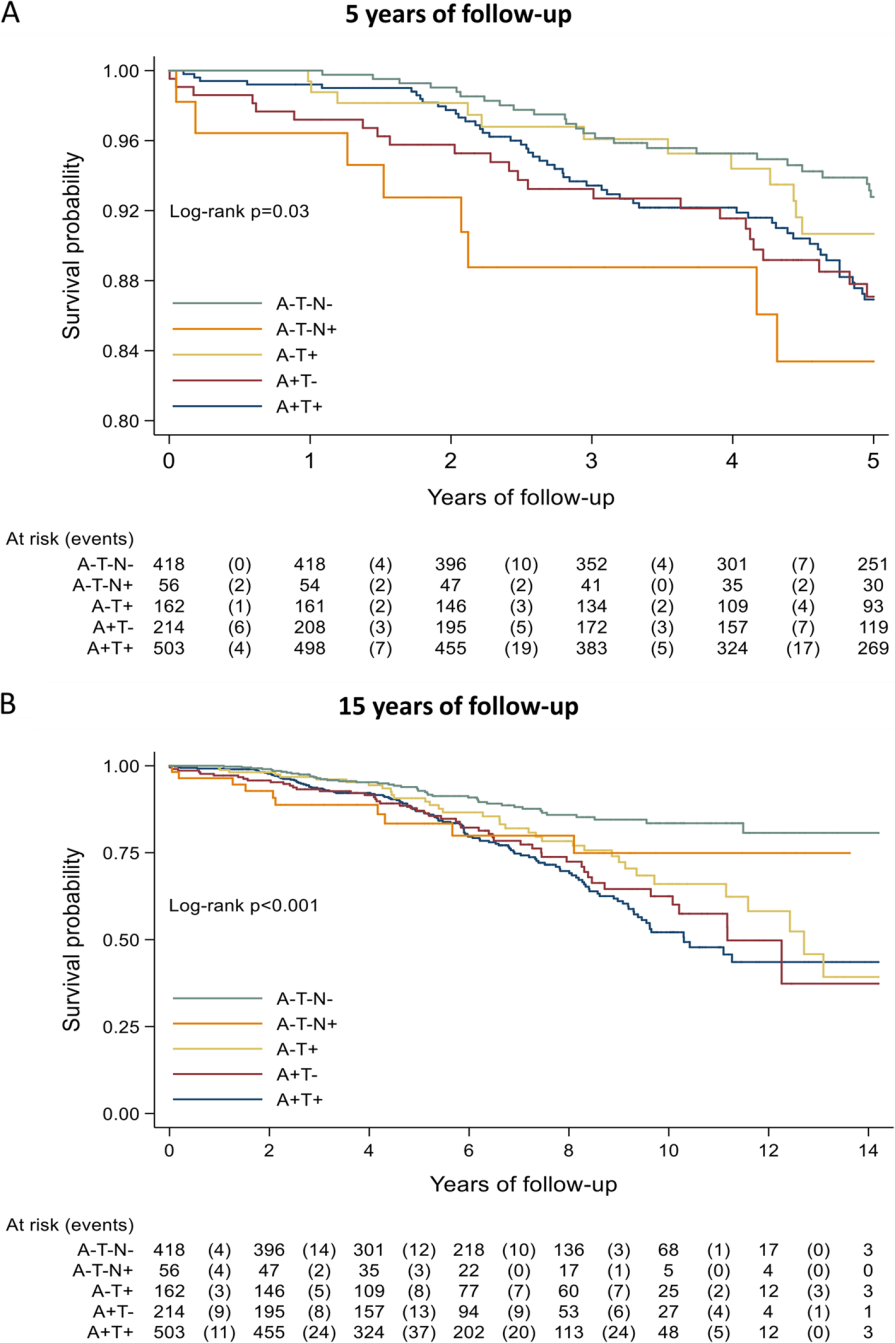
Kaplan–Meier survival estimates according to ATN profiles in the overall population over 5 (**A**) and 15 years (**B**) of follow-up

Figure 1 shows the Kaplan–Meier curves for mortality as a function of ATN profiles. At both the 5-year (*p* for log-rank = 0.03) and 15-year follow-up (*p* < 0.001), patients with normal AD biomarker (A-T −) profiles had the lowest probability of mortality. Figure 1A shows that at the 5-year follow-up, individuals with A-T-N + profile had the lowest survival probability compared to those with other profiles. At the 15-year follow-up (Fig. 1B), patients with Alzheimer’s continuum profiles (A +) and non-AD pathologic change (A-T +) had lower survival compared to those with normal biomarkers profiles, with the lowest survival probability in A + T + patients. The median survival was 10.3 years in those with the A + T + profile, 11.2 years in those with the A + T − profile, and 12.7 years in those with the A-T + profile. The survival rate was greater than 75% at the end of follow-up in both A-T-N − and A-T-N + groups, not allowing estimation of median survival in these groups.

**Fig. 2:**
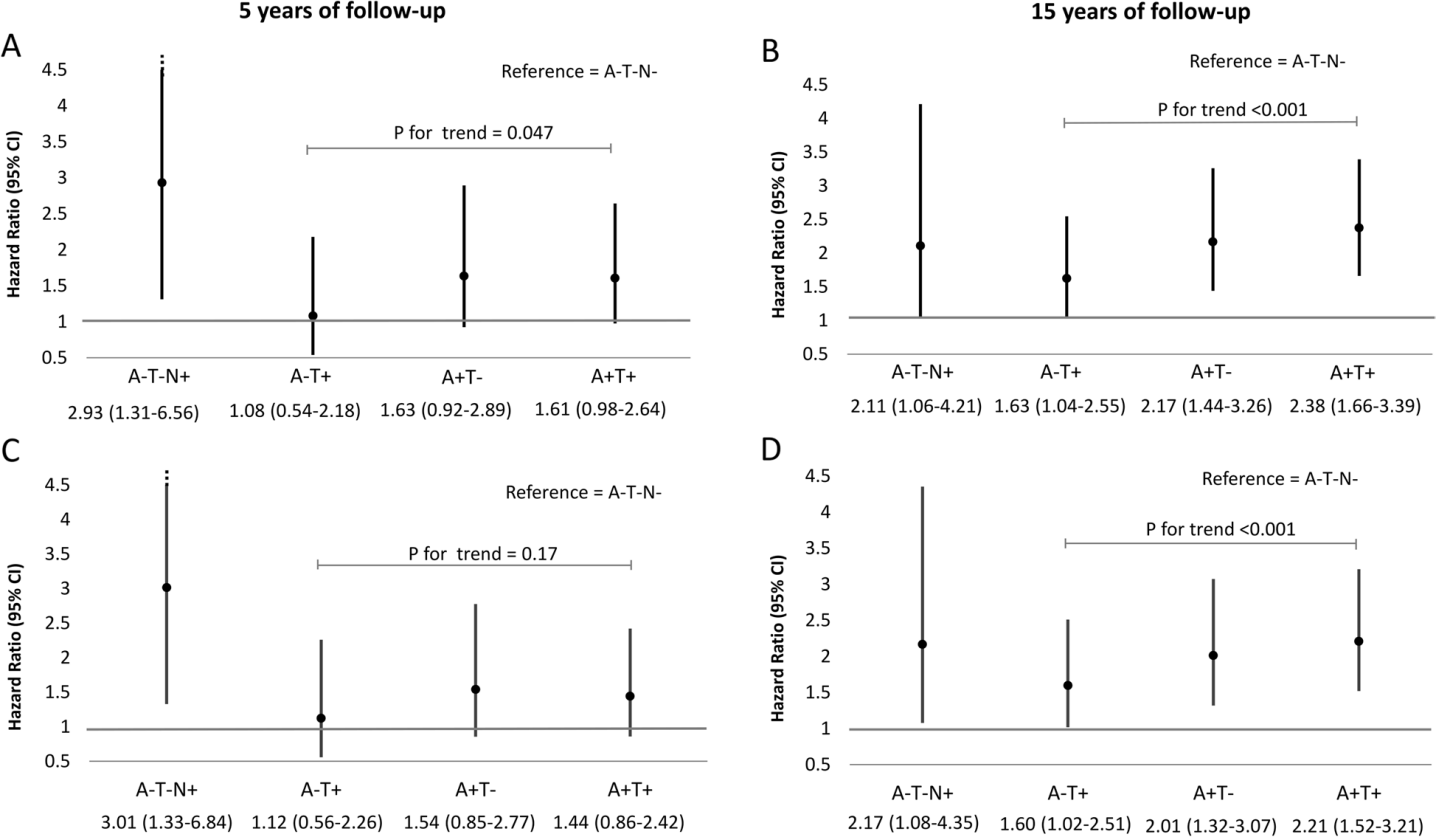
Hazard ratios (95% CI) of mortality for each ATN profile over 5 (**A**, **C**) and 15 years (**B**, **D**) of follow-up. Reference group = A-T-N − . **A**, **B** Cox model adjusted for age and sex. **C**, **D** Cox model adjusted for age, sex, APOE4 status, MMSE score, level of education, smoking status, hypertension, and dyslipidemia

The multivariable Cox regression analyses for the risk of mortality as a function of ATN profiles are shown in Fig. 2. At the 5-year follow-up, A-T-N + individuals had a near threefold higher risk of mortality (hazard ratio (HR): 2.93; 95% confidence interval (CI) 1.31–6.56) compared to A-T-N − individuals in analysis adjusted for age and sex. There was no significant association of mortality with other biomarker profiles (Fig. 2 A). At the 15-year follow-up, those with the A-T + profile (non-AD pathologic change) had a 1.6 higher risk of mortality (HR: 1.63 (95% CI 1.04–2.55)). There was a gradient along the AD stages with HRs (95% CI) for mortality of 2.17 (1.44–3.26) for A + T- patients and 2.38 (1.66–3.39) for A + T + patients (*p* for trend < 0.001, Fig. 2 B). Further adjustment for APOE4 status, MMSE score, level of education, smoking status, hypertension, and dyslipidemia had little impact on these estimates (Fig. 2C, D).

### Impact of sex on the relationship between ATN profiles and mortality risk

There was no significant difference between men and women in the association between ATN profile and mortality risk (*p* for interaction = 0.65); analyses stratified on sex are shown in Supplementary Figure S1.

### Impact of cognitive stage on the association between ATN profiles and mortality risk

There was no evidence that cognitive stage influenced the association between ATN profile and long-term mortality risk (*p* for interaction = 0.30); analyses stratified on the cognitive stage are shown in Supplementary Table S4. The A + T + profile was associated with mortality irrespective of the cognitive stage.

### CSF biomarkers of AD and risk of mortality

**Fig. 3:**
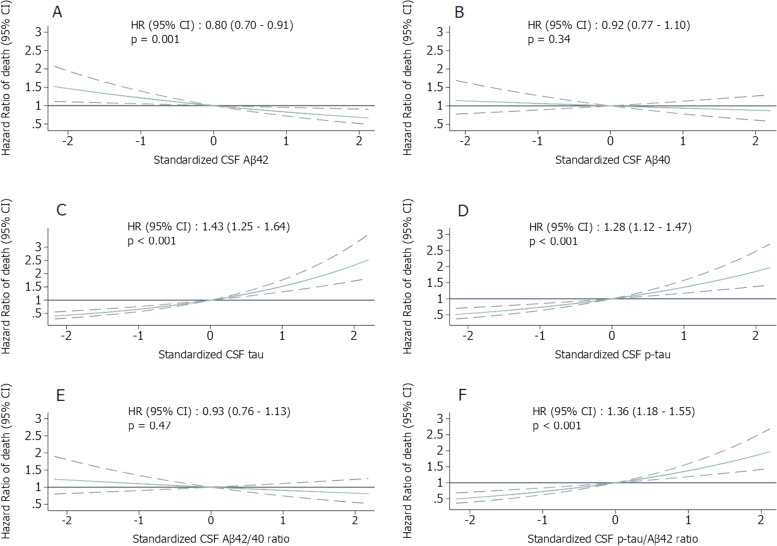
Hazard ratios of mortality according to CSF AD biomarkers. Dotted lines indicate the 95% confidence interval. Cox models adjusted for APOE4 status, age, sex, MMSE score, level of education, smoking status, hypertension, and dyslipidemia

Figure 3 shows the association between each CSF biomarker and mortality. In models adjusted for age, sex, APOE4, level of education, MMSE score, and cardiovascular risk factors, a 1 SD increase in CSF level of Aβ42 was associated with a 20% lower risk of mortality (HR: 0.80 (95% CI 0.70–0.91), *p* = 0.001, Fig. 3A); a 1 SD increase in CSF levels of tau, CSF p-tau181, and CSF p-tau181/Aβ42 ratio was associated with a 43% (HR: 1.43 (95% CI 1.25–1.64), Fig. 3C), 28% (HR: 1.28 (95% CI 1.12–1.47), Fig. 3D), and 36% (HR: 1.36 (95% CI 1.18–1.55), Fig. 3F) higher risk of mortality respectively. Among those with measures of CSF Aβ40 (*N* = 980), there was no association between CSF Aβ40 and mortality (*p* = 0.34).

## Discussion

This study on the association between CSF biomarker-based ATN profiles and mortality in a relatively large sample of memory clinic patients presents 3 key findings. One, there was a strong association between ATN profiles and mortality in the longer follow-up, with a twofold higher risk in A + T + patients compared to those with a normal A-T-N − profile, with the median survival being 10.3 years in persons with the A + T + profile. Two, in the shorter follow-up (5 years), patients with isolated markers of neurodegeneration (A-T-N +) had the higher risk of mortality with the highest risk observed in the initial period and subsequent stabilization over time. Three, all CSF biomarkers considered individually were also associated with mortality in the long follow-up, with the strongest association seen with CSF tau. This highlights the role of markers of neurodegeneration in the overall prognosis of cognitive disorders.

We aimed to investigate the association between ATN profiles and mortality in a large sample of patients seen at a memory clinic for cognitive disorders. The fact that Alzheimer’s disease is the 5th leading cause of death among older adults in developed countries highlights the importance of understanding the impact of this disease on mortality. The novelty of our study is the use of CSF biomarkers for a biological definition of AD rather than clinical criteria alone. The use of biomarkers allows AD definition to be uniform across all individuals and reduces the potential bias for false positives due to other causes of dementia such as vascular disease or Lewy body disease. Our findings showing reduced median survival associated with an A + T + profile (10.3 years) highlight the need for effective interventions for individuals with this profile. When disease-modifying drugs for Alzheimer’s disease become available, it would be important to study whether treatments extend survival in patients with this biomarker profile.

Few studies have previously examined the association between biomarkers in the ATN classification and mortality. A previous study in a population of patients with Lewy body dementia showed that those within the AD continuum had a steeper cognitive decline and shorter survival [24]. Two studies have also reported an association of brain amyloidopathy assessed on PET amyloid [25] or neurodegeneration assessed by brain MRI atrophy with a higher risk of mortality [25, 26]. Our results showed an increased risk of mortality in patients in the Alzheimer’s continuum (A + T − , A + T +) and patients with non-AD pathologic change (A-T + , A-T-N +) compared to patients with normal AD biomarkers (A-T-N −). When CSF AD biomarkers were examined separately, CSF tau was the most strongly associated with mortality. Studies that examined the association between individual CSF biomarkers of AD and mortality have shown contrasting results; some have reported an association with CSF tau [18, 27] and hyperphosphorylated tau [17], while others did not find an association [18, 27, 28]. As in the present study, most studies found a lower level of CSF Aβ42 to be associated with a higher risk of mortality [18, 28, 29].

The present study adds to the current literature by showing the length of the follow-up to affect associations. Isolated neurodegeneration (A-T-N +) in our study was associated with a higher risk of mortality at the 5-year follow-up compared to patients with normal AD biomarkers profiles. This may be explained by the fact that isolated markers of neurodegeneration may detect acute events in the brain (stroke, meningitis, epilepsy…) that affect survival in the short term. For example, it has been reported that mortality risk following ischemic stroke is particularly high during the first 5 years post-stroke [30, 31]. The usefulness of “neurodegeneration” status in the ATN classification remains the subject of debate, leading the “N” to be included in brackets in the AT(N) classification. Our results argue for the importance of determining the “neurodegeneration” status in the biomarker profiles of patients by showing its prognostic role for mortality in the short term.

We also found a higher risk of mortality among patients with suspected non-amyloid pathology (A-T +) as compared to patients with normal AD biomarkers. Among patients with suspected non-amyloid pathology, some may have tauopathies such as progressive supranuclear palsy (PSP) or frontotemporal dementia (FTD) [32]. Mortality in PSP [33, 34] is thought to depend on age at onset, with older age at onset associated with higher mortality risk [35] and quick disease progression, in terms of the appearance of symptoms [35]. Survival after diagnosis of FTD varies between 4.5 and 9.1 years [36].

Patients in the Alzheimer’s continuum in our study were also more likely to die within the 15-year follow-up compared to patients with normal AD biomarkers. The median survival time among AD patients using clinical diagnostic criteria was reported to be 5.8 years in a recent literature review [37], justifying our choice of 5 years in the short-term follow-up. To further investigate the impact of this cutoff on our findings, we calculated the association between ATN profile and short-term mortality using different time-frames for the follow-up: 3 years, 4 years, 5 years, and 6 years (Supplementary Table S5). Interestingly, the shorter the follow-up time, the stronger the association of the A-T-N + profile with mortality, which underlines our hypothesis of an association of this profile with short-term mortality. In the present study, we found a median survival time of 10.3 years for A + T + patients. Differences could be explained by potential earlier diagnosis of AD in memory clinics due to the use of CSF biomarkers. We also found a gradient in mortality risk according to AD stage with a greater risk of mortality in A + T + compared to A + T − patients. Although the APOE ε4 allele is a major genetic risk factor in non-sporadic AD and is associated with higher mortality risk [38], we found no impact of APOE4 on the association between ATN classification and risk of mortality. These findings suggest that while APOE4 increases the risk of AD, it does not play a role in shaping mortality risk. These findings need confirmation in studies based on a larger number of cases.

## Limitations

The main strengths of the study are the relatively large sample size with over 1300 patients to enable analyses in various ATN subgroups, the use of CSF biomarkers to characterize the ATN profiles, and the completeness of data on the vital status of each participant due to linkage to mortality data in France. The limitations of the study include the lack of data on the cause of death, limiting the analyses to all-cause mortality. A further limitation is that the study is based on data from memory centers where consultations aim to determine the cause of reported cognitive complaints, and results based on this population may not be generalizable to other population settings, particularly the general population. Despite the relatively large size of our sample of patients, some ATN profiles were rare, not allowing us to examine some of the sub-groups in detail.

## Conclusion

In conclusion, this study on the association between ATN profiles and risk of mortality in a large population of patients from memory clinics showed a short-term higher risk of mortality in patients with isolated evidence of neurodegeneration (A-T-N +). In the long-term, patients in the AD continuum (A +) showed the highest risk of mortality, but median survival was longer than in studies based on clinical criteria of AD, highlighting the importance of biomarkers for early identification of AD.

## Supplementary Information

**Additional file 1:** **Supplementary Table S1.** Evolution of CSF biomarkers’ cut-offs. **Supplementary Table S2.** Association between “N” status and all-cause mortality risk in analyses stratified by “AT” categories. **Supplementary Table S3.** Characteristics of the study population by ATN profiles. **Supplementary Table S4.** Association between ATN profiles and mortality risk stratified by cognitive stage. **Supplementary Table S5.** Association between ATN profiles and short-term mortality using different time-frames. **Supplementary Figure S1.** Association between A/T/N profiles and mortality according to sex.

## Data Availability

The dataset generated and analyzed in the current study is available from the corresponding author upon reasonable request.

## Abbreviations

AD: Alzheimer’s disease
ATN: β-Amyloid (A), tau lesion (T), neurodegeneration (N)
Aβ: β-Amyloid peptide
PET: Positron emission tomography
CSF: Cerebrospinal fluid
p-tau181: Phosphorylated tau
NIA-AA: National Institute on Aging – Alzheimer’s Association
MMSE: Mini-Mental State Examination
APOE: Apolipoprotein E
HR: Hazard ratio
CI: Confidence intervals
PSP: Supranuclear palsy
FTD: Frontotemporal dementia

## References

[1] GBD 2016 Dementia Collaborators (2019). Global, regional, and national burden of Alzheimer’s disease and other dementias, 1990–2016: a systematic analysis for the Global Burden of Disease Study 2016. Lancet Neurol.

[2] GBD 2019 Dementia Forecasting Collaborators (2022). Estimation of the global prevalence of dementia in 2019 and forecasted prevalence in 2050: an analysis for the Global Burden of Disease Study 2019. Lancet Public Health.

[3] Alzheimer’s Association Report (2021). 2021 Alzheimer’s disease facts and figures. Alzheimers Dement.

[4] Hyman BT, Phelps CH, Beach TG (2012). National Institute on Aging-Alzheimer’s Association guidelines for the neuropathologic assessment of Alzheimer’s disease. Alzheimers Dement.

[5] Blennow K, Mattsson N, Scholl M, Hansson O, Zetterberg H (2015). Amyloid biomarkers in Alzheimer’s disease. Trends Pharmacol Sci.

[6] Cohen AD, Landau SM, Snitz BE (2019). Fluid and PET biomarkers for amyloid pathology in Alzheimer’s disease. Mol Cell Neurosci.

[7] Brier MR, Gordon B, Friedrichsen K (2016). Tau and Abeta imaging, CSF measures, and cognition in Alzheimer’s disease. Sci Transl Med..

[8] Jack CR, Bennett DA, Blennow K (2018). NIA-AA Research Framework: toward a biological definition of Alzheimer’s disease. Alzheimers Dement..

[9] Dubois B, Villain N, Frisoni GB (2021). Clinical diagnosis of Alzheimer’s disease: recommendations of the International Working Group. Lancet Neurol.

[10] Delmotte K, Schaeverbeke J, Poesen K, Vandenberghe R (2021). Prognostic value of amyloid/tau/neurodegeneration (ATN) classification based on diagnostic cerebrospinal fluid samples for Alzheimer’s disease. Alzheimers Res Ther.

[11] Ingala S, De Boer C, Masselink LA (2021). Application of the ATN classification scheme in a population without dementia: findings from the EPAD cohort. Alzheimers Dement.

[12] Ebenau JL, Visser D, Kroeze LA (2022). Longitudinal change in ATN biomarkers in cognitively normal individuals. Alzheimers Res Ther.

[13] Tan MS, Ji X, Li JQ (2020). Longitudinal trajectories of Alzheimer’s ATN biomarkers in elderly persons without dementia. Alzheimers Res Ther.

[14] Carandini T, Arighi A, Sacchi L (2019). Testing the 2018 NIA-AA research framework in a retrospective large cohort of patients with cognitive impairment: from biological biomarkers to clinical syndromes. Alzheimers Res Ther.

[15] Burnham SC, Coloma PM, Li QX (2019). Application of the NIA-AA research framework: towards a biological definition of Alzheimer’s disease using cerebrospinal fluid biomarkers in the AIBL study. J Prev Alzheimers Dis.

[16] Calvin CM, de Boer C, Raymont V (2020). Prediction of Alzheimer’s disease biomarker status defined by the ‘ATN framework’ among cognitively healthy individuals: results from the EPAD longitudinal cohort study. Alzheimers Res Ther.

[17] Degerman Gunnarsson M, Lannfelt L, Ingelsson M, Basun H, Kilander L (2014). High tau levels in cerebrospinal fluid predict rapid decline and increased dementia mortality in Alzheimer’s disease. Dement Geriatr Cogn Disord.

[18] Boumenir A, Cognat E, Sabia S (2019). CSF level of beta-amyloid peptide predicts mortality in Alzheimer’s disease. Alzheimers Res Ther.

[19] Lafirdeen ASM, Cognat E, Sabia S (2019). Biomarker profiles of Alzheimer’s disease and dynamic of the association between cerebrospinal fluid levels of β-amyloid peptide and tau. PLoS ONE.

[20] Paquet C, Bouaziz-Amar E, Cognat E (2018). Distribution of cerebrospinal fluid biomarker profiles in patients explored for cognitive disorders. J Alzheimers Dis.

[21] Dumurgier J, Sabia S, Zetterberg H, et al. Pragmatic, Data-driven method to determine cutoffs for CSF biomarkers of Alzheimer disease based on validation against PET imaging*.* Neurology. 2022;99(7):e669–78. 10.1212/WNL.0000000000200735. 10.1212/WNL.0000000000200735 PMC9484605 35970577

[22] Jack CR, Bennett DA, Blennow K (2016). A/T/N: an unbiased descriptive classification scheme for Alzheimer disease biomarkers. Neurology..

[23] Poli M, Gatta LB, Dominici R (2005). Apolipoprotein E haplotyping by denaturing high-performance liquid chromatography. Clin Chem Lab Med.

[24] van de Beek M, Ooms FAH, Ebenau JL (2022). Association of the ATN research framework with clinical profile, cognitive decline, and mortality in patients with dementia with Lewy bodies. Neurology.

[25] Lopez OL, Becker JT, Chang Y (2018). Amyloid deposition and brain structure as long-term predictors of MCI, dementia, and mortality. Neurology.

[26] van der Veen PH, Muller M, Vincken KL (2014). Brain volumes and risk of cardiovascular events and mortality. The SMART-MR study. Neurobiol Aging.

[27] Rhodius-Meester HFM, Liedes H, Koene T (2018). Disease-related determinants are associated with mortality in dementia due to Alzheimer’s disease. Alzheimers Res Ther.

[28] Wallin AK, Blennow K, Andreasen N, Minthon L (2006). CSF biomarkers for Alzheimer’s disease: levels of beta-amyloid, tau, phosphorylated tau relate to clinical symptoms and survival. Dement Geriatr Cogn Disord.

[29] Ribbe M, Kern S, Borjesson Hansson A (2019). Amyloid beta42 and total tau levels in cerebrospinal fluid associate with survival in an 85-year-old population-based cohort followed until death. Dement Geriatr Cogn Disord.

[30] Rutten-Jacobs LC, Arntz RM, Maaijwee NA (2013). Long-term mortality after stroke among adults aged 18 to 50 years. JAMA.

[31] Bravata DM, Ho SY, Brass LM (2003). Long-term mortality in cerebrovascular disease. Stroke.

[32] Bang J, Spina S, Miller BL (2015). Frontotemporal dementia. Lancet.

[33] Williams DR, Lees AJ (2009). Progressive supranuclear palsy: clinicopathological concepts and diagnostic challenges. Lancet Neurol.

[34] Holmberg B, Johnels B, Blennow K, Rosengren L (2003). Cerebrospinal fluid Aβ42 is reduced in multiple system atrophy but normal in Parkinson’s disease and progressive supranuclear palsy. Mov Disord.

[35] Arena JE, Weigand SD, Whitwell JL (2016). Progressive supranuclear palsy: progression and survival. J Neurol.

[36] Coyle-Gilchrist IT, Dick KM, Patterson K (2016). Prevalence, characteristics, and survival of frontotemporal lobar degeneration syndromes. Neurology.

[37] Liang CS, Li DJ, Yang FC (2021). Mortality rates in Alzheimer’s disease and non-Alzheimer’s dementias: a systematic review and meta-analysis. Lancet Healthy Longev.

[38] Appiah D, Baumgartner RN (2018). The influence of education and apolipoprotein epsilon4 on mortality in community-dwelling elderly men and women. J Aging Res.

