# Supplementary material for: Association between ATN profiles and mortality in a clinical cohort of patients with cognitive disorders
Source: Alzheimers Res Ther. 2023 Apr 10;15:77. doi: 10.1186/s13195-023-01220-x (PMC10088112; doi:10.1186/s13195-023-01220-x)

**Supplementary material**

**Supplementary Table S1**. Change in CSF biomarkers’ cut-offs over time

| Periods  (dd/mm/yy) | Cut-offs | | | |
| --- | --- | --- | --- | --- |
|  | aβ42, pg/mL | t-tau, pg/mL | p-tau, pg/mL | aβ42/40 ratio |
| 01/01/08 | 500 | 300 | 65 | 0.071 |
| 05/12/12 | 815 | 300 | 58 | 0.065 |
| 20/05/16 | 730 | 340 | 58 | 0.083 |
| 24/05/18 | 860 | 225 | 22 | 0.083 |

**Supplementary Table S2**. Association between "N" status (+ vs -) and all-cause mortality risk in analyses stratified by "AT" categories (A-T-, A+T-, A-T+, A+T+).

|  | **N** | **HR [IC]** | **P value** |
| --- | --- | --- | --- |
| A-T-N- | 418 | 1 | Ref |
| A-T-N+ | 56 | 2.22 [1.10 - 4.46] | 0.03 |
|  |  |  |  |
| A-T+N- | 16 | 1 | Ref |
| A-T+N+ | 146 | 1.76 [0.40 - 7.87] | 0.46 |
|  |  |  |  |
| A+T-N- | 151 | 1 | Ref |
| A+T-N+ | 63 | 1.13 [0.60 - 2.11] | 0.71 |
|  |  |  |  |
| A+T+N- | 14 | 1 | Ref |
| A+T+N+ | 489 | 0.78 [0.25 - 2.46] | 0.67 |
|  |  |  |  |

Cox models adjusted for age, sex, and level of education.

**Supplementary Table S3**. Characteristics of the study population by ATN profiles.

|  |  |  |  |  |  |  |  |  |  |  |  |
| --- | --- | --- | --- | --- | --- | --- | --- | --- | --- | --- | --- |
|  |  |  | A/T/N profiles | | | | | | | |  |
|  | Overall population |  | A-/T-/N- | A-/T-/N+ | A-/T+/N- | A-/T+/N+ | A+/T-/N- | A+/T-/N+ | A+/T+/N- | A+/T+/N+ |  |
|  | N= 1353 |  | N = 418 | N = 56 | N = 16 | N = 146 | N = 151 | N = 63 | N = 14 | N = 489 | P value |
| Age, mean (SD) | 68.2 (9.8) |  | 63.8 (10.9) | 66.1 (9.1) | 64.8 (8.0) | 71.3 (8.0) | 68.5 (9.3) | 71.8 (9.5) | 70.6 (5.5) | 70.6 (8.3) | <0.001 |
| Women, n (%) | 722 (53.4) |  | 193 (46.2) | 33 (58.9) | 9 (56.3) | 75 (51.4) | 74 (49.0) | 35 (55.6) | 7 (50.0) | 296 (60.5) | 0.004 |
| Education level, n (%) |  |  |  |  |  |  |  |  |  |  |  |
| Low | 370 (27.4) |  | 103 (24.6) | 9 (16.1) | 4 (25.0) | 43 (29.5) | 46 (30.5) | 18 (28.6) | 6 (42.9) | 141 (28.8) |  |
| Intermediate | 455 (33.6) |  | 146 (34.9) | 20 (35.7) | 5 (31.3) | 52 (35.6) | 52 (34.4) | 18 (28.6) | 5 (35.7) | 157 (32.1) |  |
| High | 528 (39.0) |  | 169 (40.4) | 27 (48.2) | 7 (43.8) | 51 (34.9) | 53 (35.1) | 27 (42.9) | 3 (21.4) | 191 (39.1) | 0.637 |
| MMSE score, mean (SD) | 22.6 (5.4) |  | 24.5 (4.1) | 23.3 (5.8) | 25.4 (3.7) | 23.6 (4.5) | 22.4 (5.7) | 19.8 (6.7) | 19.7 (6.8) | 21.0 (5.7) | <0.001 |
| APOE, n (%) |  |  |  |  |  |  |  |  |  |  |  |
| e2/e2 | 4 (0.3) |  | 1 (0.3) | 0 | 0 | 2 (1.4) | 1 (0.8) | 0 | 0 | 0 |  |
| e2/e3 | 96 (8.0) |  | 49 (13.3) | 8 (16.7) | 1 (7.1) | 8 (5.8) | 3 (2.4) | 4 (7.1) | 1 (7.1) | 22 (5.0) |  |
| e2/e4 | 25 (2.1) |  | 3 (0.8) | 2 (4.2) | 0 | 2 (1.4) | 3 (2.4) | 1 (1.8) | 0 | 14 (3.2) |  |
| e3/e3 | 594 (49.3) |  | 244 (66.1) | 29 (60.4) | 6 (42.9) | 85 (61.2) | 58 (46.0) | 16 (28.6) | 7 (50.0) | 149 (33.9) |  |
| e3/e4 | 387 (32.1) |  | 70 (19.0) | 8 (16.7) | 6 (42.9) | 38 (27.3) | 52 (41.3) | 30 (53.6) | 3 (21.4) | 180 (41.0) |  |
| e4/e4 | 99 (8.2) |  | 2 (0.5) | 1 (2.1) | 1 (7.1) | 4 (2.9) | 9 (7.1) | 5 (8.9) | 3 (21.4) | 74 (16.9) | <0.001 |
| APOE4 carriers, n (%) | 511 (42.4) |  | 75 (20.3) | 11 (22.9) | 7 (50.0) | 44 (31.7) | 64 (50.8) | 36 (64.3) | 6 (42.9) | 268 (61.1) | <0.001 |
| Heavy alcohol consumption, n (%) | 87 (7.6) |  | 36 (10.3) | 4 (8.9) | 0 | 7 (5.7) | 9 (7.3) | 2 (3.9) | 1 (8.3) | 28 (6.7) | 0.555 |
| Smokers, n (%) | 123 (10.5) |  | 41 (11.2) | 8 (17.0) | 3 (20.0) | 9 (7.3) | 16 (12.6) | 4 (7.6) | 1 (9.9) | 41 (9.6) | 0.471 |
| Dyslipidemia, n (%) | 387 (31.3) |  | 107 (28.1) | 13 (26.5) | 5 (33.3) | 51 (37.8) | 43 (32.3) | 9 (16.4) | 4 (33.3) | 155 (33.8) | 0.094 |
| Hypertension, n (%) | 562 (45.2) |  | 157 (40.7) | 19 (38.8) | 4 (26.7) | 70 (52.6) | 64 (47.8) | 20 (36.4) | 8 (66.7) | 220 (47.8) | 0.041 |
| Diabetes mellitus, n (%) | 191 (15.4) |  | 67 (17.5) | 8 (16.3) | 2 (13.3) | 24 (17.9) | 17 (12.8) | 6 (10.9) | 2 (16.7) | 65 (14.2) | 0.770 |
|  |  |  |  |  |  |  |  |  |  |  |  |

**Supplementary Table S4**. Association between ATN profiles and mortality risk stratified by cognitive stage.

|  |  |  |  |  |  |  |  |
| --- | --- | --- | --- | --- | --- | --- | --- |
|  |  |  | Model 1^a^ | |  | Model 2^b^ | |
| **Cognitively unimpaired** | N at risk | N death | HR [IC] | p |  | HR [IC] | p |
| A-T-N- | 155 | 3 | 1 | Ref |  | 1 | Ref |
| A-T-N+ | 17 | 3 | 3.80 [0.94 - 15.4] | 0.06 |  | 3.83 [0.92 - 15.9] | 0.07 |
| A-T+ | 53 | 7 | 2.36 (0.83 - 6.73] | 0.11 |  | 2.33 [0.79 - 6.82] | 0.12 |
| A+T- | 55 | 7 | 3.22 [1.17 - 8.85] | 0.02 |  | 3.14 [1.12 - 8.82] | 0.03 |
| A+T+ | 82 | 16 | 3.86 [1.54 - 9.66] | 0.004 |  | 4.00 [1.52 - 10.5] | 0.01 |
| **Mild Cognitive Impairment** |  |  |  |  |  |  |  |
| A-T-N- | 184 | 28 | 1 | Ref |  | 1 | Ref |
| A-T-N+ | 26 | 4 | 1.66 [0.56 - 4.86] | 0.36 |  | 1.56 [0.52 - 4.69] | 0.43 |
| A-T+ | 66 | 21 | 1.67 [0.92 - 3.06] | 0.09 |  | 2.02 [1.08 - 3.76] | 0.03 |
| A+T- | 73 | 21 | 1.87 [1.01 - 3.45] | 0.047 |  | 2.20 [1.14 - 4.24] | 0.02 |
| A+T+ | 189 | 46 | 1.76 [1.06 - 2.92] | 0.03 |  | 2.03 [1.17 - 3.54] | 0.01 |
| **Dementia** |  |  |  |  |  |  |  |
| A-T-N- | 79 | 13 | 1 | Ref |  | 1 | Ref |
| A-T-N+ | 13 | 3 | 2.57 [0.70 - 9.36] | 0.15 |  | 2.41 [0.65 - 8.96] | 0.19 |
| A-T+ | 43 | 8 | 0.94 [0.37 - 2.41] | 0.90 |  | 0.98 [0.38 - 2.55] | 0.97 |
| A+T- | 86 | 23 | 1.88 [0.94 - 3.74] | 0.07 |  | 2.09 [1.01 - 4.35] | 0.048 |
| A+T+ | 232 | 59 | 1.96 [1.06 - 3.63] | 0.03 |  | 2.14 [1.12 - 4.08] | 0.02 |

^a^Estimated from Cox models adjusted for age and sex.

^b^Estimated from Cox models adjusted for age, sex, APOE4 status, MMSE score, level of education, smoking, dyslipidemia, and hypertension

**Supplementary Table S5**. Association between ATN profiles and short-term mortality using different time-frames.

|  |  |  |  |
| --- | --- | --- | --- |
| **At 3 years of follow-up** | N death | HR [IC] | p value |
| A-T-N- | 14 | 1 | Ref |
| A-T-N+ | 6 | 4.38 [1.66 - 11.5] | 0.003 |
| A-T+ | 6 | 1.00 [0.38 - 2.64] | 0.994 |
| A+T- | 14 | 1.69 [0.79 - 3.62] | 0.175 |
| A+T+ | 30 | 1.67 [0.86 - 3.25] | 0.131 |
| **At 4 years of follow-up** |  |  |  |
| A-T-N- | 18 | 1 | Ref |
| A-T-N+ | 6 | 3.25 [1.28 - 8.27] | 0.013 |
| A-T+ | 8 | 1.07 [0.46 - 2.49] | 0.882 |
| A+T- | 17 | 1.67 [0.85 - 3.30] | 0.137 |
| A+T+ | 35 | 1.59 [0.88 - 2.90] | 0.126 |
| **At 5 years of follow-up** | |  |  |
| A-T-N- | 25 | 1 | Ref |
| A-T-N+ | 8 | 2.93 [1.31 - 6.56] | 0.009 |
| A-T+ | 12 | 1.08 [0.54 - 2.18] | 0.823 |
| A+T- | 24 | 1.63 [0.92 - 2.89] | 0.092 |
| A+T+ | 52 | 1.61 [0.98 - 2.64] | 0.062 |
| **At 6 years of follow-up** |  |  |  |
| A-T-N- | 30 | 1 | Ref |
| A-T-N+ | 9 | 2.79 [1.32 - 5.92] | 0.007 |
| A-T+ | 16 | 1.21 [0.65 - 2.24] | 0.543 |
| A+T- | 30 | 1.77 [1.06 - 2.96] | 0.03 |
| A+T+ | 72 | 1.92 [1.23 - 2.99] | 0.004 |
|  |  |  |  |

Cox models adjusted for age and sex.

**Supplementary Figure S1**. Association between ATN profiles and mortality risk stratified by sex. Analysis is adjusted for age.

Reference = A-T-N-


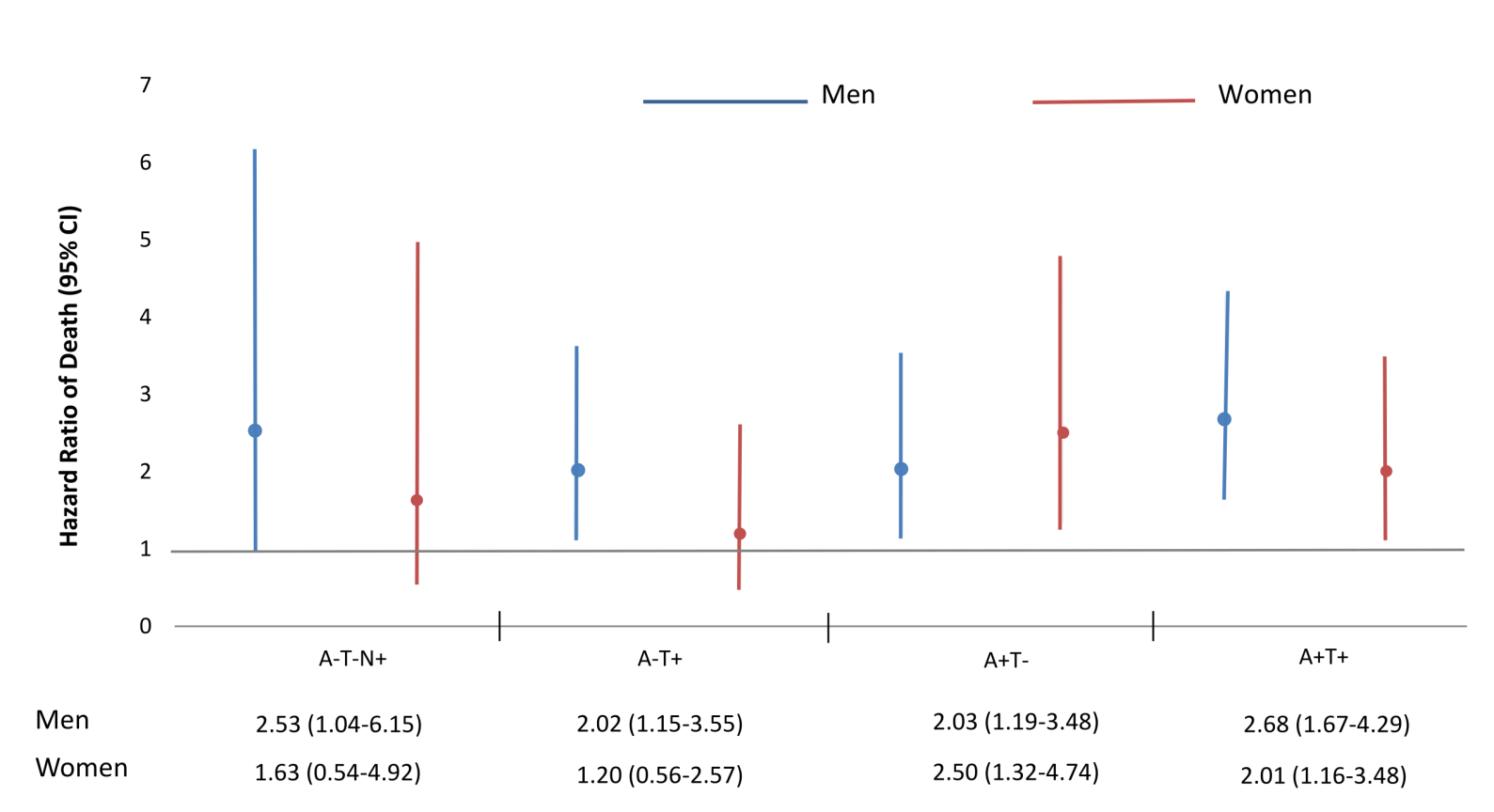

Supplement: Supplementary file 1 — Additional file 1: Supplementary Table S1. Evolution of CSF biomarkers’ cut-offs. Supplementary Table S2. Association between “N” status and all-cause mortality risk in analyses stratified by “AT” categories. Supplementary Table S3. Characteristics of the study population by ATN profiles. Supplementary Table S4. Association between ATN profiles and mortality risk stratified by cognitive stage. Supplementary Table S5. Association between ATN profiles and short-term mortality using different time-frames. Supplementary Figure S1. Association between A/T/N profiles and mortality according to sex. [file 13195_2023_1220_MOESM1_ESM.docx]
